# Current State of and Future Opportunities for Prediction in Microbiome Research: Report from the Mid-Atlantic Microbiome Meet-up in Baltimore on 9 January 2019

**DOI:** 10.1128/mSystems.00392-19

**Published:** 2019-10-08

**Authors:** Eric Sakowski, Gherman Uritskiy, Rachel Cooper, Maya Gomes, Michael R. McLaren, Jacquelyn S. Meisel, Rebecca L. Mickol, C. David Mintz, Emmanuel F. Mongodin, Mihai Pop, Mohammad Arifur Rahman, Alvaro Sanchez, Winston Timp, Jeseth Delgado Vela, Carly Muletz Wolz, Joseph P. Zackular, Jessica Chopyk, Seth Commichaux, Meghan Davis, Douglas Dluzen, Sukirth M. Ganesan, Muyideen Haruna, Dan Nasko, Mary J. Regan, Saul Sarria, Nidhi Shah, Brook Stacy, Dylan Taylor, Jocelyne DiRuggiero, Sarah P. Preheim

**Affiliations:** a Department of Environmental Health and Engineering, Johns Hopkins University, Baltimore, Maryland, USA; b Department of Biology, Johns Hopkins University, Baltimore, Maryland, USA; c Molecular and Comparative Pathobiology, Johns Hopkins University, Baltimore, Maryland, USA; d Department of Earth and Planetary Sciences, Johns Hopkins University, Baltimore, Maryland, USA; e Department of Population Health and Pathobiology, North Carolina State University, Raleigh, North Carolina, USA; f Center for Bioinformatics and Computational Biology, University of Maryland, College Park, Maryland, USA; g American Society for Engineering Education, Washington, DC, USA; h Department of Anesthesiology and Critical Care Medicine, Johns Hopkins University School of Medicine, Baltimore, Maryland, USA; i University of Maryland School of Medicine, Institute for Genome Sciences, Baltimore, Maryland, USA; j Department of Computer Science, George Mason University, Fairfax, Virginia, USA; k Department of Ecology and Evolutionary Biology, Yale University, New Haven Connecticut, USA; l Department of Biomedical Engineering, Johns Hopkins University, Baltimore, Maryland, USA; m Department of Civil and Environmental Engineering, Howard University, Washington, DC, USA; n Center for Conservation Genomics, Smithsonian National Zoological Park & Conservation Biology Institute, Washington, DC, USA; o Department of Pathology and Laboratory Medicine, University of Pennsylvania and Children’s Hospital of Philadelphia, Philadelphia, Pennsylvania, USA; p School of Public Health, University of Maryland, College Park, Maryland, USA; q Department of Environmental Health and Engineering, Johns Hopkins Bloomberg School of Public Health, Baltimore, Maryland, USA; r Department of Biology, Morgan State University, Baltimore, Maryland, USA; s National Institute of Dental and Craniofacial Research, National Institutes of Health, Bethesda, Maryland, USA; t University of Maryland School of Nursing, Baltimore, Maryland, USA; University of California San Diego

**Keywords:** microbiome, bioinformatics, metagenomics, prediction, machine learning, conceptual models, quantitative models

## Abstract

Accurate predictions across multiple fields of microbiome research have far-reaching benefits to society, but there are few widely accepted quantitative tools to make accurate predictions about microbial communities and their functions. More discussion is needed about the current state of microbiome analysis and the tools required to overcome the hurdles preventing development and implementation of predictive analyses. We summarize the ideas generated by participants of the Mid-Atlantic Microbiome Meet-up in January 2019. While it was clear from the presentations that most fields have advanced beyond simple associative and descriptive analyses, most fields lack essential elements needed for the development and application of accurate microbiome predictions. Participants stressed the need for standardization, reproducibility, and accessibility of quantitative tools as key to advancing predictions in microbiome analysis. We highlight hurdles that participants identified and propose directions for future efforts that will advance the use of prediction in microbiome research.

## Abstract (precis)

Accurate predictions across multiple fields of microbiome research have far-reaching benefits to society, but there are few widely accepted quantitative tools to make accurate predictions about microbial communities and their functions. More discussion is needed about the current state of microbiome analysis and the tools required to overcome the hurdles preventing development and implementation of predictive analyses.

## PERSPECTIVE

Prediction can be used for prognosis, interventions, and knowledge generation, since testing predicted versus actual outcomes can reveal gaps in knowledge. Therefore, developing an understanding of microbial processes to support predictions has far-reaching benefits in diverse areas of microbiome research. Advances toward predictive analyses across a spectrum of disciplines have been made, such as predicting disease states from microbial community composition (e.g., see reference 1) and environmental conditions from microbial community composition (e.g., see reference 2). Yet, the field of medicine has not routinely implemented microbiome analysis as part of standard clinical care, diagnosis, or treatment. Nor does the field of environmental engineering use microbial community composition to predict the ecosystem response to changing environmental conditions. Although there have been a few papers that have attempted to provide a framework for advancing the field of predictive analysis in microbiome research (3–5), further discussion is required to spark innovation and drive quantitative and predictive analysis forward in all microbiome research areas.

To address the topic Predictions and the Microbiome and promote additional discussions around predictive analysis, we organized the Mid-Atlantic Microbiome Meet-up (M^3^) 2019 meeting at Johns Hopkins University. M^3^ was started in 2016 at the University of Maryland College Park (spearheaded by Todd Treangen and Mihai Pop) and has spurred discussion and interactions on a variety of topics of general interest to microbiome research, including biodefense and pathogen detection (6) and metagenomic software validation (7). M^3^ conferences have also provided an excellent venue to catalyze interactions across clinical, environmental, and computational microbiome fields. As such, this meeting facilitated the presentation and discussion of issues that are broadly applicable across microbiome fields to encourage interactions and discussions of common challenges. This report summarizes the talks, posters, and breakout session discussions from this conference. We offer recommendations based on these discussions for advancing predictions in microbiome research.

## MEETING DEMOGRAPHICS

**FIG 1:**
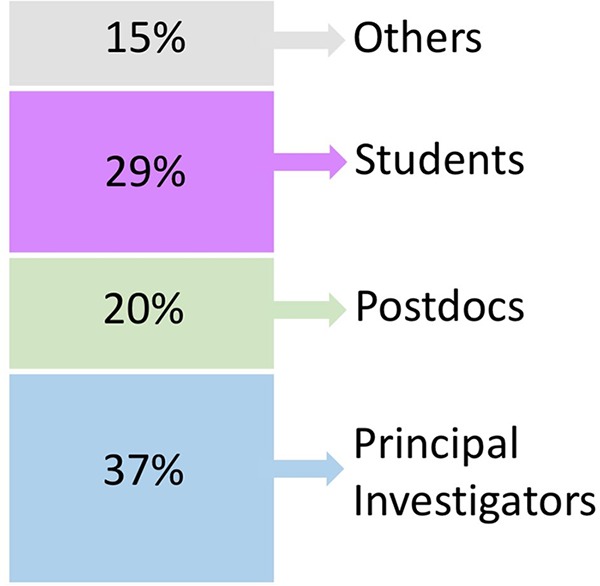
Self-reported positions of registered M^3^ attendees for the 2019 meeting.

**FIG 2:**
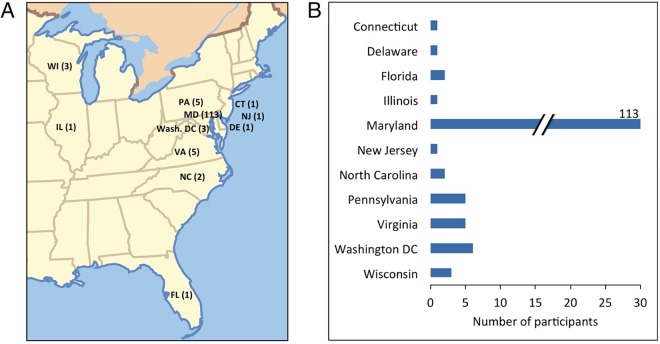
Geographic distribution of M^3^ participants drawn from registration information.

**FIG 3:**
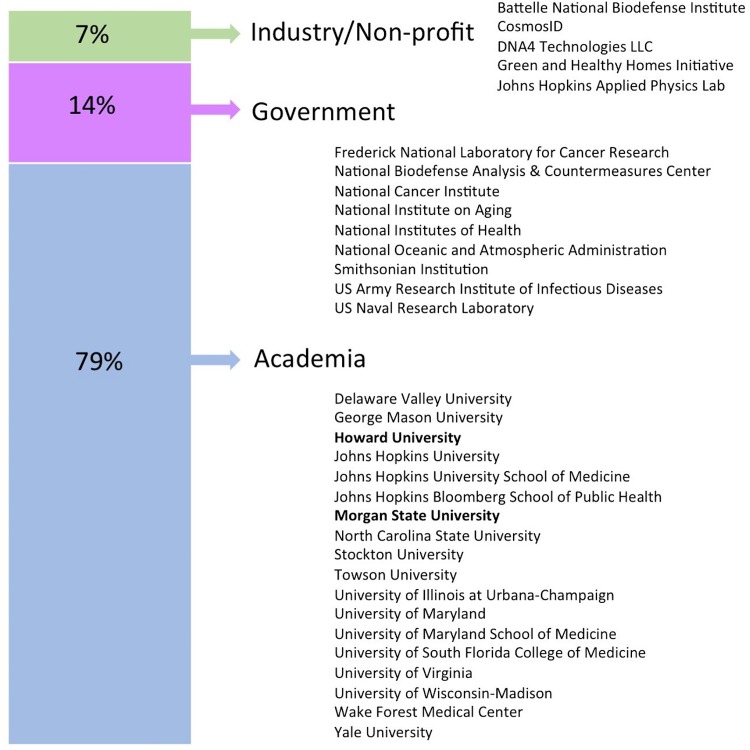
Breakdown of M^3^ participant affiliation with institutions listed.

This year’s M^3^ meeting participants represented faculty, postdocs, and students drawn largely from academic institutions around Maryland, with a minority of participants coming from farther away and from nonacademic institutions. The composition of participants was fairly evenly split between students, postdocs, and faculty, with a smaller proportion of attendees with other positions (Fig. 1). Geographically, attendees were largely drawn from the local community of Johns Hopkins University and the University of Maryland’s multiple campus locations (Fig. 2 and 3). However, M^3^ participants came from over 30 different institutions, mostly along the East Coast, including two Historically Black Colleges and Universities (HBCUs), Howard University and Morgan State University. This conference, which is local in nature, provides a venue for interactions between researchers working in the Mid-Atlantic region and familiarizes everyone with resources and expertise in their local community. One of the objectives of M^3^ is to encourage student participation by maintaining low registration and travel costs. Although this information was not collected, people from a variety of backgrounds participated in the meeting and discussions, such as environmental science, microbiology, and veterinary and human medicine, along with many participants who mentioned that they were not microbiologists or do not routinely work on the microbiome.

## DEFINITIONS AND SCOPE

We loosely define microbiome research as the analysis of composition, function, and biotic and abiotic interactions of microorganisms in complex communities. For the purpose of this discussion, participants were told to define microbiome predictions as the estimation of a set of unknown variables (from the past, present, or future) from a set of known variables, where microorganisms or their components, functions, or consequences are either the known or unknown variable. We uniquely focused on aspects of microbiome predictions that are not well-established, especially those predicting complex community structure and function at a future state, and drew on microbiology and other fields as references for examples of predictions that are well-developed and commonly implemented.

Discussions were designed to encompass a broad set of predictions stemming from existing models and modeling tools that vary with respect to their purpose, scope, implementation and methodology. As our goal was to focus on the microbiome, relevant models involve or incorporate data from multiple microbial species or groups, typically through the use of metagenomic analysis. Machine learning/statistical approaches and mechanistic/theory-based models are two broad categories of predictions that formed the basis for discussions at this conference (Table 1). Machine learning/statistical approaches (e.g., random forest, neural networks) are well suited for classification predictions from metagenomic data, but cannot always elucidate the mechanisms structuring the community and outcomes. Mechanistic/theory-based models (e.g., biogeochemical models, genome-scale metabolic models) provide a mechanistic explanation for observations typically based on theoretical underpinning, but typically lack resolution to explain a majority of metagenomic observations, especially in complex communities. The divide between models explaining observations and models testing theory was a central theme in our conference, resulting in a call to unite these approaches.

**TABLE 1:** Examples of predictive models and tools guiding conference presentations and discussions

Category	Model type or tool^*a*^	Microbiome type	Input for prediction^*b*^	Prediction
Machine-learning/statistical	Random forest (32)	Human microbiome, gut	Genus-level OTUs	Disease state for 10 different diseases
	Artificial-neural network, Bayesian network (33, 34)	Aquatic, marine, western English Channel	Environmental conditions or satellite data	Relative abundance of taxa (order level) or their associated metabolites in space or time
	Gradient boosting regression (35)	Human microbiome, gut	Meal content, daily activity, physiological features, questionnaire responses, and microbiome features	Postmeal glycemic response
	Naive Bayes and neural network models (36)	Human microbiome, gut	Informative OTUs	Colon polyps
	BSI risk index (37)	Human microbiome, gut	Pretreatment fecal OTUs	Risk of bacteremia during chemotherapy treatment
	Multiple regression on distance matrices (38)	Terrestrial, dust	Soil and climate variables	Bacterial and fungal composition
	Linear and nonlinear regression (39)	Aquatic, marine, global ocean	Environmental factors	Taxa and diversity distributions
	Regression (40)	Terrestrial, soil	Historical and contemporary climate variables	Diversity and taxon abundance as soil communities equilibrate with current conditions

Mechanistic/theory-based	Individual- or agent-based model (41)	Engineered systems, wastewater treatment	Initial state conditions, biomass growth, death and chemical reactions	Granule solute and microbial community composition
	MacArthur's resource competition model with by-product secretion and metabolic “families” (8)	Experimental, model system	Composition carbon source, microbes with randomly assigned resource uptake rates	Family-level convergence with species-level diversity on same growth media
	Global ocean circulation, biogeochemistry and ecosystem model (42–44)	Aquatic, marine, global ocean	Global ocean state, stochastically assigned microbial growth preferences or genome composition	Microbial community structure, diversity, and gene distribution
	Water column hydrological and biogeochemical model (45, 46)	Aquatic, freshwater lake	Initial chemical state, biogeochemical reactions	Microbial functional type distribution and chemical composition of future or unobserved state
	Constraint-based genome-scale metabolic model (47)	Experimental, coculture (sulfate reducer/ methanogen)	Genome-scale biogeochemical reactions, externally supplied chemicals	Acetate and methane fluxes
	Constraint-based genome-scale metabolic model (48)	Human microbiome, gut	Genome-scale biogeochemical reactions, externally supplied chemicals	Changes of metabolites in response to diet interventions

a The model type or tool is represented by the microbiome example, with the associated reference(s) in parentheses.

b Not training, if applicable.

## MEETING PRESENTATIONS: STATE OF THE FIELD

Numerous talks and posters highlighted progress in advancing microbiome research beyond a purely descriptive science by incorporating quantitative analysis or predictions. Alvaro Sanchez (Yale University) began the conference by providing an excellent framework for advancing predictions in microbiome research by focusing on how progress will likely be made when observational data can be combined with theory developed from simple, well-constrained model systems. Dr. Sanchez used the field of astronomy as an example. He highlighted how the movement of stars and planets (observational data) was combined with the variation in pendulum motion at different locations of the planet (well-constrained model system) to integrate the concept of gravity into a quantitative theory of mechanics. This knowledge built an understanding of our solar system that led to the successful deployment of probes to distant celestial bodies (e.g., the New Horizons spacecraft’s fly-by of Ultima Thule). Therefore, to advance predictions in microbiome research, we need not only observational studies, research on model systems, and theoretical concepts, but also mechanisms to bridge the gap between observation and theory. Talks at the meeting showcased research in both observational data set collection and analysis and theory development with model systems.

### Controlled experiments with model systems.

Several talks focused on the analysis of well-controlled, model systems. Dr. Sanchez described research in his lab aimed at developing a predictive, quantitative theory of microbial community assembly. Together with his team, he focuses on predicting the composition of complex natural communities under simplified and controlled growth conditions. With glucose as the sole carbon source, they found that community assembly is deterministic at the family level, with a predictable ratio of *Enterobacteriaceae* to *Pseudomonadaceae* (8), or more generally fermenters and nonfermenters, of 75% to 25%, respectively. To determine whether other carbon sources also have deterministic assembly at the family level, they used flux balance analysis to create a similarity metric to compare carbon substrates. This allowed them to cluster sugars and sugar alcohols together, separately from carboxylic acids and organic alcohols. While the 75/25 ratio of fermenters to nonfermenters held across sugars and sugar alcohols, it did not hold for carboxylic acids and organic alcohols. Nevertheless, the fermenters-to-nonfermenters relationship was robust enough to predict the family-level composition of novel carbon sources with reasonable accuracy.

Other talks highlighted research using model systems to understand basic principles of microbial community function and dynamics. Joseph Zackular (University of Pennsylvania and Children’s Hospital of Philadelphia) described his research, which uses a combination of mouse models and *in vitro* systems to study Clostridium difficile infection. Dr. Zackular described how excess dietary zinc modulates microbial community composition in mice and exacerbates C. difficile*-*associated disease (9). Furthermore, he showed how microbiota-pathogen interactions impact C. difficile infection. Using *in vitro* systems to study interspecies interactions, his work demonstrated that *Enterococcus* and C. difficile cross talk results in alterations in C. difficile growth and virulence. Dr. Zackular postulates that co-occurrence of C. difficile with certain pathogenic and commensal members of the microbiota may impact the severity of disease.

Additional talks focused on manipulating specific variables within a variety of model systems and experimental conditions. Emmanuel Mongodin (Institute for Genome Sciences, University of Maryland School of Medicine) presented work on the influence of the gut microbiome on host immunity resulting in organ transplant rejection in mice. Fecal transplants were used to manipulate the microbiome in mice receiving a heart transplant. It was found that transplanting the microbiome of pregnant mice increased survival over microbiome transplants from normal or colic mice, with the presence of Bifidobacterium pseudolongum populations identified as significantly different between these groups (10). Transplanting *Bifidobacteria* alone improved transplant outcome and altered the host immune response. Carly Muletz Wolz (Smithsonian National Zoological Park & Conservation Biology Institute) investigated the effect of temperature on the microbiome and fungal pathogen load (Batrachochytrium dendrobatidis) of salamanders. Dr. Muletz Wolz found that temperature directly and indirectly impacted salamander skin microbiome diversity, both in a predictable manner. Temperature indirectly impacted microbiome diversity through its direct effect on pathogen load, with increasing pathogen load leading to increased changes in the microbiome. Dr. Muletz Wolz also quantified how effective culturable bacterial strains were at killing *B. dendrobatidis* across different temperatures to better predict which strains could serve as probiotics across a range of temperatures.

### Observational data analysis.

Other talks were largely focused on using observations to advance our understanding and predictive power of microbial community dynamics and function of natural systems. Jacquelyn Meisel (University of Maryland) analyzed human stool microbiomes in a large case study of diarrheal disease in young children. In 60% of cases, a single pathogen could not be detected (11), suggesting that the disease state was either caused by a novel pathogen or by the association of multiple organisms. The study found over 1,000 operational taxonomic units (OTUs) enriched in cases versus controls, with Lactobacillus ruminis associated with nondysenteric samples. Facultative anaerobes were largely enriched in disease cases, including an association of *Streptococcus* with disease, which was only recently identified. In a totally different field that exemplified the diversity of the M^3^ meeting, Eric Sakowski (Johns Hopkins University) presented work to develop a novel experimental technique to identify virus infections of cyanobacteria in the ocean using emulsion paired-isolation concatenation PCR (epicPCR). This technique will fill the void in observational data that currently exists in viral ecology and is an important first step toward advancing predictions of how viruses will impact natural microbial communities. Rachel Cooper (Johns Hopkins School of Medicine) presented her work to identify whether microbiome community compositions were different in marmoset populations with and without wasting syndrome. Preliminary data suggested that the microbial communities are different, providing a mechanism to probe the cause of mortality in these animals. Finally, Rebecca Mickol (American Society for Engineering Education, U.S. Naval Research Laboratory) presented observations of the evolution of microbial communities in alternating-current (cathodic/anodic) microcosms. Metagenomic observations suggested that a number of dominant microorganisms could play a role in the generation or utilization of current.

Some talks advanced observational data analysis by attempting to develop or explain observations with conceptual or quantitative models. Gherman Uritskiy (Johns Hopkins University) presented a conceptual model framework with which to interpret shifts in the microbial community after a catastrophic rain event in salt rock communities in the Atacama Desert, Chile. The initial response to the rain was characterized by rapid shifts in both community membership and gene composition, while the microbiome’s recovery was achieved through gradual changes in the newly restructured community. These dynamics resulted in rock communities that performed similar functions to those from before the rain but were comprised of a new set of individual strains. These observations allowed for the inference of a conceptual model of community dynamics in response to perturbation and the proposal of a quantification method for the strain composition flux. Jeseth Delgado Vela (Howard University) presented work using a quantitative biogeochemical model to explain nitrogen cycling in a membrane-aerated biofilm reactor with increasing concentrations of influent sulfide. Predictions of ammonia levels from a one-dimensional biofilm model of biogeochemical processes fell short of observed amounts. Through rate experiments using ^15^N and metagenomic analysis, Dr. Vela identified dissimilatory nitrate reduction to ammonia (DNRA) coupled to sulfide or methane oxidation as a potential process to explain discrepancies between experimental results and model predictions.

Two additional talks described quantitative frameworks to interpret metagenomic data through improvements in machine learning techniques or by quantifying the bias inherent in marker gene or shotgun metagenomic analysis. Mohammad Arifur Rahman (George Mason University) discussed a multiple instance learning (MIL) framework to determine clinical outcomes based on metadata and oral, skin, and gut microbiome analyses (12). Validation and the biological significance of their approach remain to be addressed. Michael McLaren (North Carolina State University) discussed how to measure and correct the error that occurs in measurements of microbial communities by marker gene and shotgun metagenomics sequencing as each step in an experimental workflow is biased toward detecting some taxa over others. Dr. McLaren presented results of a recent study (13) that shows that a simple mathematical model captures how bias distorts community measurements of defined bacterial communities (i.e., cellular mock communities). This model can be used to quantify and correct bias (calibration) when calibration standards containing the taxa of interest are available. It can also be used to determine whether certain metagenomics-based analysis and prediction algorithms are expected to be more or less likely to be confounded by bias.

## BREAKOUT REPORT

Participants were encouraged to discuss and share their own ideas about how to use and advance predictions in their own fields of microbiome research and to identify benefits of and obstacles to implementation. A number of themes emerged from these discussions, including (i) the need for reproducibility and standards of analysis, (ii) more communication between researchers in different fields to better share ideas, and (iii) integration of observations with theory to make predictions.

### Standardization and reproducibility.

Most discussion groups mentioned the challenge for prediction posed by the quantitative incomparability of microbiome measurements made by different experiments. This was emphasized by one of the participants who remarked that, for stool microbiome analysis to be used in diagnosis and treatment, the results should not vary depending on which testing laboratory is used. Microbiome measurements, such as marker gene and metagenomics sequencing, from different experiments are typically made with a variety of sample extraction, library preparation, and sequencing methodologies, each of which lead to experiment-specific differences in the resulting measurements. A predictive model developed from data generated in one experiment may not make reliable predictions on data obtained by a different experiment that used, for example, different primer sets (14), sample storage techniques (15), or DNA extraction methods (16). In addition to such technical variation, biological differences in the cohorts or environments being sampled can result in a model that makes accurate predictions on the samples it was developed on but that do not transfer to a new set of samples. To be practically applicable, quantitative predictions will have to explain observations across multiple data sets and be able to provide useful information about the limitations and uncertainty associated with the prediction due to technical, as well as biological, variables.

A major obstacle to developing and implementing predictions in microbiome research lies in the limited use of control material (i.e., standards) and standardized protocols in microbiome experimental and analysis techniques. Standardization of protocols and widespread analysis of control materials were necessary to coordinate large-scale microbiome sequencing projects, such as the Human Microbiome Project (17, 18) and the Earth Microbiome Project (19). These efforts have also resulted in a push for more standardization, especially in the human microbiome field (http://www.microbiome-standards.org). Yet, outside of these coordinated efforts, standardization of protocols and the regular use and reporting of control material could be substantially improved, making it easier to test or validate predictions from samples collected and analyzed from different groups.

### Enhancing communication.

Additional avenues of communication are needed between researchers in different fields. It is essential to bridge the gap between the theory and tools developed for observation-based data sets and those developed for model systems through effective communication between groups. This applies to model system developers and observational data collectors, computational scientists and clinicians, human and environmental microbiome researchers, microbiologists, and even nonmicrobiologists. Observational researchers could then have access to more tools for analysis to identify relationships within their own system. Researchers may become aware of existing models and standards in other fields of study that are applicable to their own system. Proper communication requires common and basic language, the creation of conceptual models that can be used to communicate complex relationships, and mechanisms for nonexperts to access and use quantitative models developed by experts. The power and limitations of any relationship or quantitative association should also be clearly communicated, such as the extent of testing with different systems, populations, or conditions. Ideally, if a prediction or model is to be used by a specific group (e.g., doctors, engineers, or managers), people from that group should be involved in its development. These objectives are being increasingly achieved through multidisciplinary collaborations and open access to data and analysis pipelines.

### Improving predictions with machine learning techniques guided by theory.

Machine learning techniques can be used to identify previously unknown relationships, but are heavily dependent on the quality of the input data set. Machine learning allows for robust quantitation of relationships between microbial components and metadata categories even before there is a mechanistic understanding to explain the relationship. This is similar to the example from astronomy that was presented in the introductory talk. A quantitative relationship of the movement of planets based on the size of celestial bodies was developed before the role of gravity was understood. This so-called “black box” top-down approach has been most commonly applied in the context of single studies (e.g., references 20 and 21) and less often applied across multiple, uncoordinated studies (e.g., references 22 and 23). The widespread use of standard protocols and processing of control materials in microbiome analysis could allow these techniques to provide more broadly comparable data sets to enhance and strengthen predictions.

The results of machine learning techniques should be used with caution, ideally paired with biological knowledge of the system to validate the results. Machine learning techniques can identify predictive features that are not directly associated with the condition or response itself. For example, machine learning can lead to misleading predictions by picking up on extraneous features or biases (e.g., see reference 24). Additionally, the results of machine learning techniques might be misleading if the input data are biased or skewed (e.g., see reference 25). While machine learning techniques can develop predictions with some degree of accuracy, it will be important to interpret the results of these predictions within the context of in-depth biological knowledge of the system to understand what predictions mean and how to apply them properly.

## CURRENT CHALLENGES AND RECOMMENDATIONS

The field of microbiome research is poised to progress from making observations of microbial communities to predicting future states. The applications of such predictions could be many and varied, from anticipating an individual’s likelihood of developing certain diseases to forecasting the location and severity of harmful algal blooms. The continued development of quantitative analyses of microbiome observations and conceptual models that probe fundamental principles of community assembly and environmental predictors will be important steps forward. Additionally, greater efforts should be made to develop intradisciplinary and transdisciplinary standards of data collection and analyses in microbiome research. Such standards should reflect the needs of multiple stakeholder groups, including target user groups (people who would make decisions based on the output from predictions). Finally, improved communication between different fields of microbiome research would promote the spread of ideas that may be more developed in one field than another. Here, we provide recommendations based on our discussions for integrating these components toward future predictive models.

### Implementing standard protocols and control material to reduce biases and improve comparative analyses.

The use of standard protocols and routine processing of control material will result in data sets that can easily be compared and corroborated, which is critical to improving and validating microbiome predictions. Currently many individual groups choose to use their own combination of extraction methods, library preparation methods, sequencing platforms, and internal standards or commercial mock communities. Differences in library processing at almost every step affect the resulting community composition (e.g., see references 14 to 16). This limits the extent to which we can broadly compile data sets to validate predictions. Some research groups will have a compelling reason to choose novel protocols or standards, such as for improving or developing protocols or for unique or difficult environmental samples. For researchers who are not in this position, voluntarily adopting the standard protocols and control material from a large microbiome project closest to their field of study will make data sets more comparable and valuable. There are a number of such groups to choose from with accessible protocols and control materials (e.g., the Human Microbiome Project [17, 18] and the Earth Microbiome Project [19]). With acceptance and implementation of standard protocols and control materials across multiple studies, more comparable microbiome data sets will be available to test predictions or to identify novel relationships that can be investigated further.

There are still a number of challenges in widespread use of standard protocols and control materials. Standardization begins with implementing standards in metadata collection and reporting (26), which could incur unnecessary expenses collecting information that is not relevant to the specific microbiome study. Standardizing experimental protocols will require techniques that are easy to implement and robust between labs and between users with various degrees of expertise. Differences in equipment and facilities could also hamper standardization of experimental procedures, and as such, there should be accommodations for both large and small laboratories. Although the use of even the best-designed standard protocol is not likely to eliminate differences generated across different laboratories, the use of control material prepared alongside experimental samples will help to identify inherent biases.

Along with standard protocols and processing of control materials, reproducible analysis also will be critical for broadly applying predictions. Bioinformatically reducing the bias between data sets (13) will be an important advance, as samples that have been prepared with standard protocols and control materials will still contain biases. Reproducibility in data analysis across studies may not be as critical as the implementation of standard protocols and control materials in the data generation step, since multiple analysis pipelines can be applied to the same data set. Yet, steps should be taken to facilitate the reproducible analysis of data across studies, whether with implementation software developed to enhance reproducibility, such as Docker containers (27), the Anaconda environments (28), or routine publishing of analysis code. Facilitating access to data and analysis software, such as Integrated Microbial Genomes and Microbiomes (29) and Qiita (30), will also be an important aspect of microbiome analysis standardization. These steps will facilitate reproducibility and standardization in data analysis, so different groups can apply or confirm predictions.

### Oversight committee for microbiome research.

Implementing the aforementioned recommendations for standardization could be more easily achieved if there was strong leadership by an oversight committee specifically for microbiome research. There are a number of different groups promoting the use of standards for various aspects of microbiome analysis, such as the International Human Microbiome Standards (IHMS) project (http://www.microbiome-standards.org/), the Genomic Standards Consortium (https://press3.mcs.anl.gov/gensc/), and the Association of Biomolecular Resource Facilities (https://abrf.org/research-group/metagenomics-mgrg). To be maximally effective, efforts should be coordinated through strong leadership from a federally supported oversight committee, such as the National Microbiome Initiative and groups developing microbiome standards at the National Institute of Standards and Technology. Researchers could refer to this oversight committee for field-specific protocols, control materials, new analysis tools, and training. This group could work to incentivize biotechnology companies to develop and commercialize standard protocols and control materials. While individual researchers would not be required to adhere to the recommendations of this committee, there could be pressure from funding agencies or publishing groups to develop data in compliance with standard protocols if there is not a clear need to deviate from these standards. Finally, the committee could foster dialogue between different fields and research sectors by organizing and sponsoring meetings like M^3^.

### Testing and validating microbiome predictions.

Predictions developed in model systems or with machine learning techniques need extensive validation to be widely accepted and useable. Both the microbial community composition and the environments that microorganisms inhabit are complicated and difficult to predict. Thus, it is unlikely that one model will be developed that will hold across multiple systems anytime soon, so most models will depend on the specific question being asked. This is largely due to the fact that the number of factors influencing the system is so great that no study can measure all of the relevant metadata. Because of this, the scope and applicability of any relationship should be clearly communicated, and efforts should be made to validate relationships across more systems, such as was suggested during the breakout session for clinical and experimental studies of the inflammosome (e.g., see reference 31). This may result in a simplification or generalization of any relationship with insight from additional testing. For example, Dr. Sanchez and his group were able to generalize the reproducible patterns they observed in community assembly in glucose-only media by simplifying by function (i.e., fermenters/nonfermenters) once they expanded their observations to multiple carbon sources. The act of formulating and testing predictions can often provide valuable insight into the factors that influence microbial communities.

## CONCLUSIONS

We hope to advance the use of predictions in microbiome research by summarizing the presentations and discussions held during the M^3^ 2019 meeting on Predictions and the Microbiome. Microbiome research has advanced beyond purely descriptive analyses, but there are still major hurdles preventing the development of accurate, reliable predictions for most microbiome applications. Participants highlighted the need to merge theories and principles developed from the analysis of model systems with quantitative analysis of observations from natural systems in order to advance accurate and reliable predictions. They identified the lack of reproducibility, the need for standardization, and the reduction of biases in microbiome analysis as the biggest hurdles to developing reliable predictions. Participants also felt that breaking down barriers to communication between fields and engaging diverse groups in research was the best way to make progress. Although most fields do not routinely apply microbiome predictions, the act of making predictions forces researchers to be quantitative in their analysis and tests the limits of previously identified relationships such that advances can be made. It is our hope that the power of quantitative analysis and the hurdles to implementation identified in this report will inspire and guide researchers as they work toward advancing predictive analysis in their field of microbiome research.
